# ^18^F-fluorodeoxyglucose (FDG) PET or ^18^F-fluorothymidine (FLT) PET to assess early response to aromatase inhibitors (AI) in women with ER+ operable breast cancer in a window-of-opportunity study

**DOI:** 10.1186/s13058-021-01464-1

**Published:** 2021-08-23

**Authors:** Perrin E. Romine, Lanell M. Peterson, Brenda F. Kurland, Darrin W. Byrd, Alena Novakova-Jiresova, Mark Muzi, Jennifer M. Specht, Robert K. Doot, Jeanne M. Link, Kenneth A. Krohn, Paul E. Kinahan, David A. Mankoff, Hannah M. Linden

**Affiliations:** 1 Division of Medical Oncology, University of Washington/Seattle Cancer Care Alliance, 1144 Eastlake (Mail Stop LG-200), Seattle, WA 98109-1023 USA; 2 University of Pittsburgh, Pittsburgh, PA USA; 3 Department of Radiology, University of Washington, Seattle, WA USA; 4 Department of Oncology, First Faculty of Medicine, Charles University and Thomayer Hospital, Prague, Czech Republic; 5 Department of Radiology, University of Pennsylvania, Philadelphia, PA USA; 6 Department of Diagnostic Radiology, Oregon Health and Science University, Portland, OR USA

**Keywords:** FDG-PET, FLT-PET, Ki-67, ER+ breast cancer, Aromatase inhibitors

## Abstract

**Purpose:**

This study evaluated the ability of ^18^F-Fluorodeoxyglucose (FDG) and ^18^F-Fluorothymidine (FLT) imaging with positron emission tomography (PET) to measure early response to endocrine therapy from baseline to just prior to surgical resection in estrogen receptor positive (ER+) breast tumors.

**Methods:**

In two separate studies, women with early stage ER+ breast cancer underwent either paired FDG-PET (*n* = 22) or FLT-PET (*n* = 27) scans prior to endocrine therapy and again in the pre-operative setting. Tissue samples for Ki-67 were taken for all patients both prior to treatment and at the time of surgery.

**Results:**

FDG maximum standardized uptake value (SUVmax) declined in 19 of 22 lesions (mean 17% (range −45 to 28%)). FLT SUVmax declined in 24 of 27 lesions (mean 26% (range −77 to 7%)). The Ki-67 index declined in both studies, from pre-therapy (mean 23% (range 1 to 73%)) to surgery [mean 8% (range < 1 to 41%)]. Pre- and post-therapy PET measures showed strong rank-order agreement with Ki-67 percentages for both tracers; however, the percent change in FDG or FLT SUVmax did not demonstrate a strong correlation with Ki-67 index change or Ki-67 at time of surgery.

**Conclusions:**

A window-of-opportunity approach using PET imaging to assess early response of breast cancer therapy is feasible. FDG and FLT-PET imaging following a short course of neoadjuvant endocrine therapy demonstrated measurable changes in SUVmax in early stage ER+ positive breast cancers. The percentage change in FDG and FLT-PET uptake did not correlate with changes in Ki-67; post-therapy SUVmax for both tracers was significantly associated with post-therapy Ki-67, an established predictor of endocrine therapy response.

**Supplementary Information:**

The online version contains supplementary material available at 10.1186/s13058-021-01464-1.

## Introduction

Adjuvant endocrine therapy improves outcomes for estrogen-receptor positive (ER+) breast cancer [1–3]. However, 25–50% of women with early stage breast cancer (stages I and II) will experience tumor recurrence [4]. Pre-operative or neoadjuvant 'window' studies provide short exposures to systemic therapy between cancer diagnosis and surgery, potentially providing early insight into tumor sensitivity and resistance [5–7]. Recent and ongoing trials use an early biopsy strategy to determine whether alternative treatment (such as chemotherapy) is indicated [8, 9]. Serial biopsy studies have shown a decrease in proliferative index (Ki-67) following as little as 2 weeks of successful neoadjuvant endocrine therapy [10]; in the IMPACT study, proliferation dropped at 2 weeks and remained low for the subsequent 10 weeks in the majority of patients [11]. Post-therapy Ki-67 levels following 2 weeks of neoadjuvant endocrine therapy have been shown to predict progression-free survival [12], but the requirement for biopsy, and often serial biopsies, results in limited clinical use.

As more data emerge that endocrine therapy alone is sufficient for some patients [13], tools are needed to measure tumor response to determine which patients benefit from chemotherapy or molecularly targeted therapies [13, 14]. Oncotype Dx is a tissue-based genomic assay that, obtained prior to therapy, is widely used to assign individual treatment options [13–15]. The ability to measure the impact of endocrine therapy could add value beyond pre-therapy predictions of response. PET imaging biomarkers offer a distinct and complementary approach to tissue sampling for evaluating early treatment response. Unlike genomic assays which rely on core-biopsy, PET has the potential to avoid sampling error, and to noninvasively assesses the entire tumor burden in vivo, allowing for serial studies.

^18^F-Fluorodeoxyglucose (FDG), the most commonly used PET imaging biomarker, measures glucose metabolism. FDG-PET has been shown to correlate with tumor proliferation in some studies [16], but is also associated with other processes such as inflammation, cellular repair, and apoptosis. ^18^F-Fluorothymidine (FLT) is an investigational imaging probe of tumor proliferation [17] shown to correlate with Ki-67 in breast, lung, and brain cancer [18, 19]. Both imaging agents have potential to identify endocrine sensitive tumors early in treatment and may identify patients who could avoid cytotoxic therapy and/or benefit from combination endocrine therapy [20] or endocrine therapy plus molecularly targeted agents [21].

Early changes in FDG-PET measure response to chemotherapy, endocrine therapy, and targeted therapy in breast and other tumors [22, 23]. FLT-PET imaging has demonstrated ability to measure early response to systemic endocrine, chemotherapy, radiotherapy, and combined chemoradiotherapy in multiple tumor types [21, 24]. FLT-PET is correlated with changes in tumor proliferation early after initiating second-line docetaxel chemotherapy and following completion of variable neoadjuvant chemotherapy regimens in breast cancer [19, 25]. Pre-clinical studies suggest that FLT-PET may be useful for indicating the need for combined endocrine therapy and cell-cycle targeted drugs (CDK4/6) [26].

We prospectively evaluated early response to neoadjuvant aromatase inhibitor (AI) therapy using baseline and pre-surgery FDG- and FLT-PET imaging in two different protocols, in conjunction with tissue Ki-67 assay in early-stage ER+ tumors under the hypothesis that one or both tracer imaging approaches would produce results similar to Ki-67 biopsy levels. Our goals were to use PET imaging to evaluate feasibility of the window-of-opportunity approach to assess endocrine therapy early response in breast cancer, and to measure early tumor response in order to improve treatment selection for early stage breast cancer that would provide insight into potential mechanisms of resistance to therapy using the Ki-67 assay, an established predictor of endocrine responsiveness [8, 12] as the reference standard.

## Methods

### Study design

**Fig. 1:**
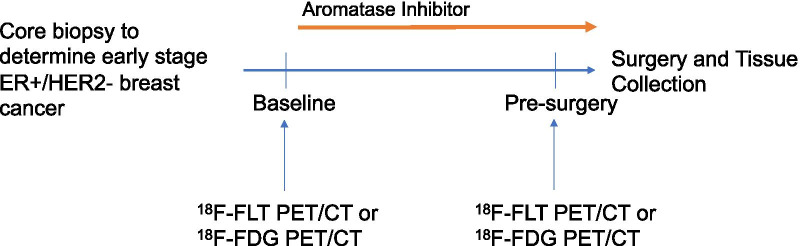
Study schema

Patients with early stage ER+ and human epithelial growth factor 2 negative (HER2-) invasive ductal or lobular breast cancer (> 1 cm) planning surgery and adjuvant endocrine therapy were eligible. Patients were enrolled from our multidisciplinary clinics between 2010–2015, following clinical diagnostic biopsy and underwent either FDG-PET/CT or FLT-PET/CT imaging, determined by tracer availability at time of consent. Permission for Ki-67 analysis of archived clinical diagnostic biopsy tissue was obtained at time of consent. Patients underwent baseline imaging and then began non-steroidal AI therapy (anastrozole or letrozole) based on treating oncologist preference. Premenopausal patients started ovarian function suppression with Gonadotropin Releasing Hormone (GnRH) agonist 2 weeks before starting AI. Following 1–9 weeks of AI ± ovarian suppression therapy (duration determined by patient preference and surgical availability), a second PET scan, on the same scanner as the initial scan, was completed prior to curative breast surgery, at which time fresh tissue was collected. Figure 1 illustrates the study schema.

### PET imaging

FDG was purchased commercially from Cardinal Health (Seattle, WA). FLT was prepared in the UW Radiochemistry service as described in Investigation New Drug (IND) #112478. Fasting was required prior to FDG-PET scan. Images were acquired dynamically over a single field-of-view, centered over the affected breast, for 60 min in the same manner as previous studies [27–30]. Summed SUV images from 30 to 60 min post-injection were constructed from the dynamic data. SUVmax was the primary uptake parameter analyzed. We chose this measure to minimize partial volume effects in these patients with relatively modest sized tumors. We analyzed the tumor that had pre-therapy tissue sampling. This was the tumor with the highest tracer uptake in those patients with multiple tumor sites. Additional file 1: Table S1 shows additional methodology, including reconstruction algorithms, and equations for other uptake measures examined.

**Table 1:** Patient characteristics

	FDG study (*N* = 22)	FLT study (*N* = 27)
	Mean (range)	Mean (range)
Age at registration, years	62.0 (51–80)	58.7 (28–74)
Days between scans	19.5 (7–40)	27.2 (6–64)
Days between 1st scan and surgery (AI therapy duration)	22.5 (9–42)	31.6 (9–69)
Days between 2nd scan and surgery	3.0 (1–6)	4.3 (1–12)*
	*N* (%)	*N* (%)
Female sex	22 (100%)	27 (100%)
Race		
Caucasian/White	19 (86%)	22 (82%)
Black/African American	–	2 (7%)
Asian	1 (5%)	3 (11%)
Pacific Islander	2 (9%)	–
Ethnicity		
Not hispanic or latino	21 (95%)	26 (96%)
Hispanic or latino	1 (5%)	1 (4%)
Menopausal status		
Postmenopausal	22 (100%)	20 (74%)
Premenopausal	–	7 (26%)
Aromatase inhibitor		
Anastrozole	18 (82%)	22 (81%)
Letrozole	4 (18%)	5 (19%)

*1 patient had 12 days between FLT scan and surgery, the remaining were 7 days or less

### Image analysis

A certified nuclear medicine physician with more than 20 years of experience used the 30–60 min summed image sets to identify the primary breast lesion used for the diagnostic biopsy. The lesion used for image analysis was the lesion where the biopsy was done. All lesions had baseline uptake in the primary tumor that exceeded background. Anatomic imaging was also available (i.e., mammography, ultrasound, (magnetic resonance imaging (MRI), and/or computed tomography (CT)) to provide information on lesion location that was correlated with position via PET and CT to localize the lesion. For post-therapy scans, patients were placed in the scanner in a position closely matching the baseline scan. In the few cases where the lesion uptake post-therapy was difficult to discern from background, the baseline scan was used as a guide for volume-of-interest (VOI) placement. Square VOIs of 3 × 3 pixels were drawn on identified lesions over three consecutive slices encompassing the pixels with the most uptake, using imaging software PMOD version 3.6 (Zurich, Switzerland) [31, 32].

### Immunohistochemistry

Clinical immunohistochemistry (IHC) was completed on biopsy tissue as part of breast cancer diagnosis, including ER status, progesterone receptor (PR) status, HER2, and Ki-67. Ki-67 was also measured on the resected tumor tissue at surgery. Ki-67 was assessed as described by Dowsett et al. and scored according to the International Ki-67 in Breast Cancer Working Group recommendations [33, 34] by certified pathologists with 10–20 years of experience.

### Statistical analysis

For each breast lesion, both the unit difference in uptake and the percentage uptake difference between the two PET scans were recorded. Metabolic response for FDG-PET was prospectively defined as a 20% decline, as this decrease would be unlikely to be due to chance [35]. Similarly, imaging response for FLT-PET was defined as a 15% decline based on a prior single institution lung study that most closely mimics this study [36].

Post-therapy Ki-67 value ≤ 10% was prospectively set as the criterion for endocrine responsiveness based on published work defining a value > 10% following AI as a marker of AI resistance [8].

Associations between tissue and imaging measures were summarized using Spearman (rank) correlations. Comparison of dichotomized PET response by Ki-67 response category used the mid-p correction to Fisher’s exact test [37] (SAS/STAT v9.4, SAS Institute, Inc., Cary, NC, USA).

## Results

### Patient characteristics

A total of 55 women were enrolled: 24 in the FDG study and 31 in the FLT study. Within the FDG study, two patients withdrew; one prior to any research procedures, and one after hospitalization for an unrelated event. Within the FLT study, four patients were removed from data analyses. One patient had tracer infiltration on her second scan, while three patients withdrew prior to any study procedures. Seven of 27 patients in the FLT study were pre-menopausal (on ovarian suppression), and 0/22 in the FDG study. Patient characteristics for the 49 evaluable patients are shown in Table 1.

### Tumor characteristics and response to endocrine therapy

**Fig. 2:**
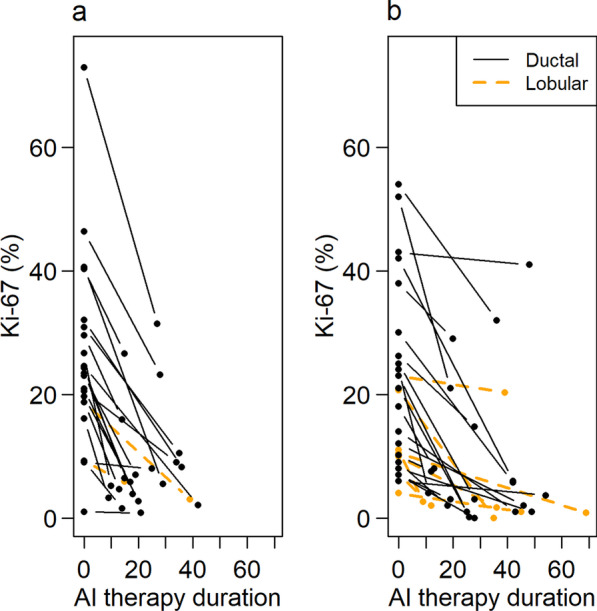
Diagnostic and surgical measures of Ki-67 index. **a** FDG study **b** FLT study shown as days on AI therapy

Table 2 shows tumor and treatment characteristics for both studies. Over the two series, baseline Ki-67 percentage ranged from 1 to 73 (mean 23); 76% (37/49) of samples had a pre-therapy value > 10%. Ki-67 percentage decreased following AI therapy in all cases (mean 15%, range 0–42%) (Fig. 2). At surgery, 22% (11/49) of all surgical samples had a Ki-67 value > 10%.

**Table 2:** Tumor and treatment characteristics

	FDG study (*N* = 22)		FLT study (*N* = 27)	
	*N* (%)	Mean (range)	*N* (%)	Mean (range)
Diagnosis				
Invasive ductal	20 (91%)		20 (74%)	
Invasive lobular	2 (9%)		7 (26%)	
Tumor size (cm)		2.6 (0.7–7.5)		3.1 (1.1–7.6)*
T Stage				
T1	9 (41%)		8 (30%)	
T2	12 (55%)		15 (56%)	
T3	1 (5%)		3 (11%)	
X	–		1 (4%)	
ER+	22 (100%)		27 (100%)	
PR+	20 (91%)		22 (81%)	
Her2-	22 (100%)		26 (96%)^†^	
Ki-67 (Biopsy)				
All		25.4 (1.0–72.9)		20.6 (4–54)
Ductal		26.5 (1–72.9)		23.5 (6–54)
Lobular		14 (9.2–18.8)		12.3 (4–23)
Ki-67 (at surgery)				
All		8.7 (1.0–31.4)		7.9 (0–41)
Ductal		9.1 (< 1.0–31.4)		9.3 (0–41)
Lobular		4.4 (3–5.9)		4.1 (0–20.3)
Ki-67 ≤ 10% (at surgery)				
All	17 (77%)		21 (78%)	
Ductal	15 (75%)		15 (75%)	
Lobular	2 (100%)		6 (86%)	

*Tumor sized by MRI in all but one patient. That patient had a lesion size of 1.1 cm by contrast enhanced CT

^†^1 patient was scored as HER2 equivocal, but no plan for treatment change

### Imaging results

**Fig. 3:**
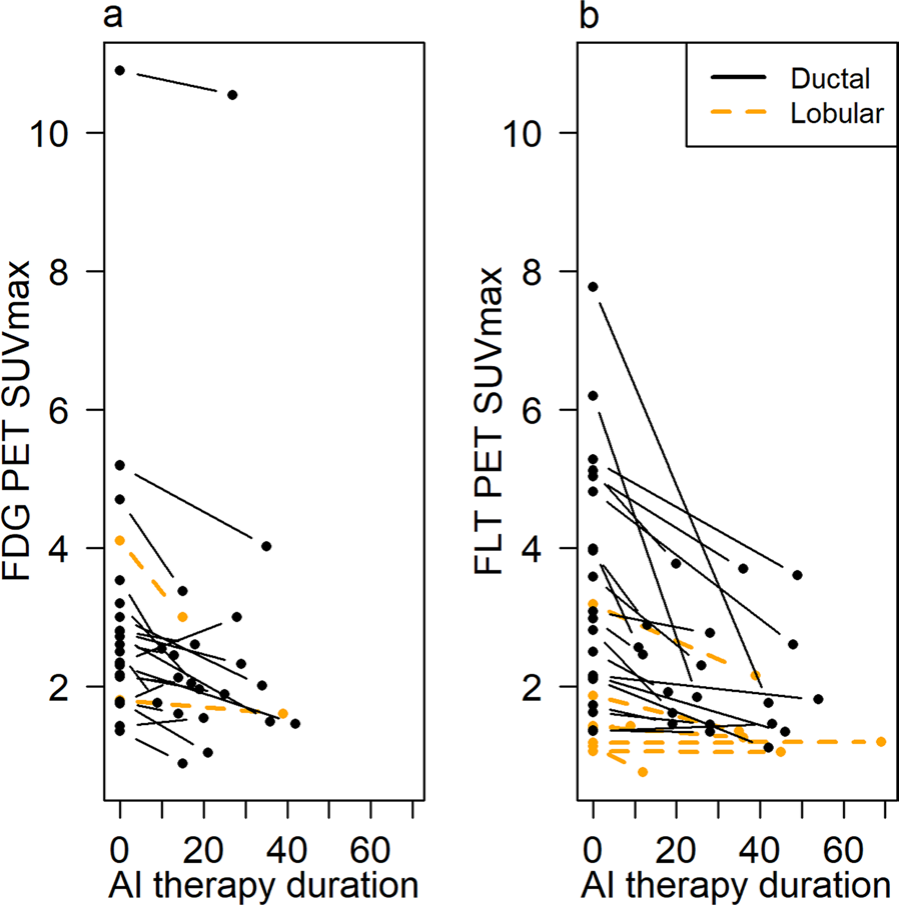
Pre-treatment and post-treatment measures **a** FDG SUVmax **b** FLT SUVmax shown as days on AI therapy

**Fig. 4:**
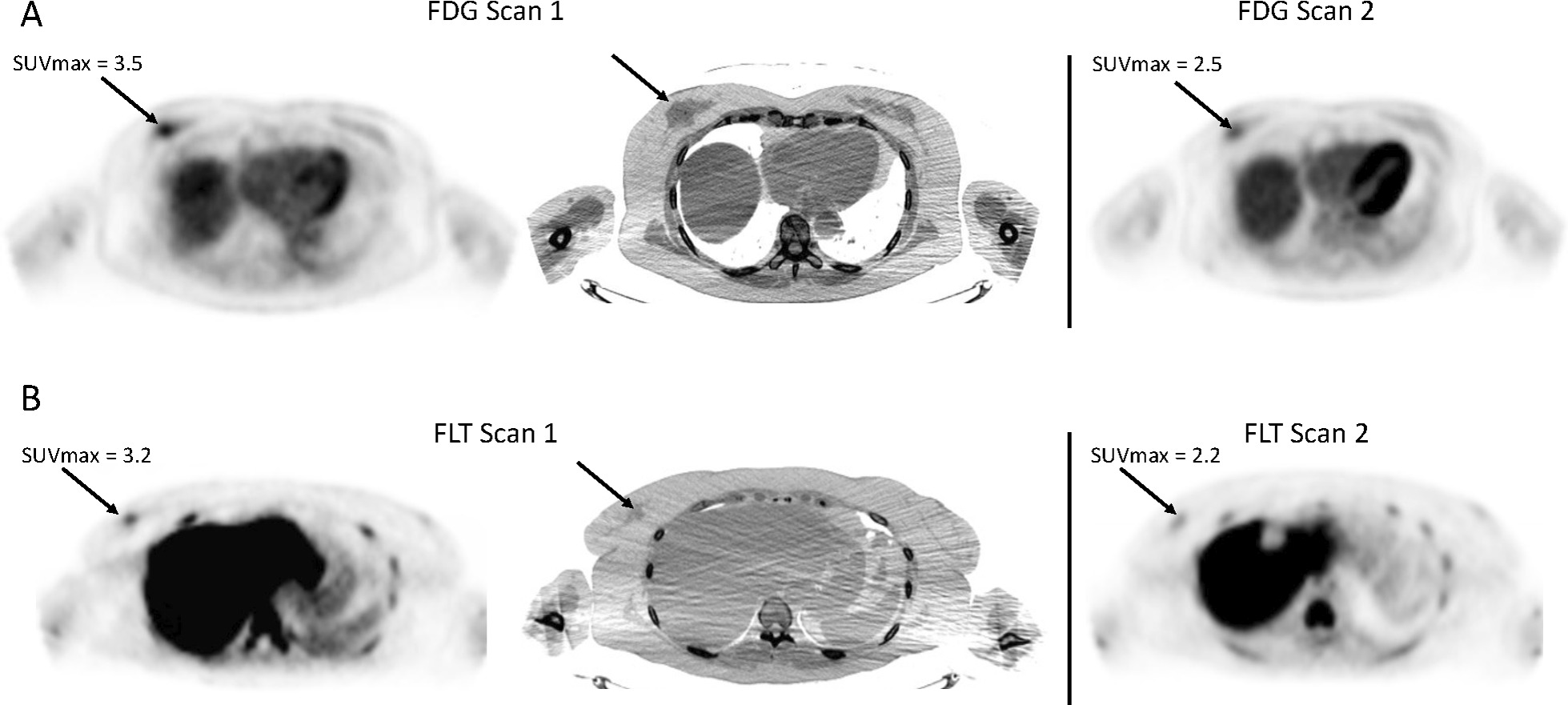
Representative early FDG and FLT-PET responses. Top panel: 72-year-old female with invasive ductal carcinoma at diagnosis. 7 days between FDG scans. Ki-67 went from 27 to 5%. SUVmax reduced from 3.5 to 2.5 at the time of follow up FDG-PET. Bottom panel: 57-year-old female with invasive lobular carcinoma. 35 days between FLT scans. Ki-67 went from 23 to 20%. SUVmax reduced from 3.2 to 2.2 at the time of follow-up FLT-PET. CTs for attenuation are shown for imaging baseline

Table 3 shows descriptive statistics for SUVmax for both studies, with graphical display in Fig. 3. Baseline FDG SUVmax was generally low, with only 4/22 (18%) values greater than 4. A decline in SUVmax by > 20% occurred for 50% (11/22) of patients in the FDG study (Fig. 3a). Baseline FLT SUVmax was also generally low with a mean of 3.0 (range 1.1–7.8). A decrease in FLT uptake occurred for 70% (19/27) of patients in the FLT study with a mean of − 26% (range − 77 to 7%) (Fig. 3b). The median time between the baseline and the second PET scan was 19.5 (range 7–40) days for FDG and 27.2 (range 6–64) days for FLT. Figure 4 shows characteristic imaging responses.

**Table 3:** FDG and FLT-PET imaging results

PET measure	FDG (*N* = 22)	FLT (*N* = 27)
	Mean (range)	
SUVmax (pre-therapy)		
All	3.0 (1.4–10.9)	3.0 (1.1–7.8)
Ductal	3.1 (1.4–10.9)	3.5 (1.4–7.8)
Lobular	3.0 (1.8–4.1)	1.6 (1.1–3.2)
SUVmax (post-therapy)		
All	2.5 (0.9–10.6)	2.0 (0.8–3.8)
Ductal	2.5 (0.9–10.6)	2.2 (1.1–3.8)
Lobular	2.3 (1.6–3.0)	1.3 (0.8–2.2)
SUVmax (percent change)		
All	−17% (−45 to 28%)	−26% (−77 to 7%)
Ductal	−17% (−45 to 28%)	−30% (−77 to 7%)
Lobular	−19% (−27 to −11%)	−15% (−33 to 1%)
SUVmax (unit change)		
All	−0.5 (−1.3 to 0.7)	−1.0 (−6.0 to 0.1)
Ductal	−0.5 (−1.3 to 0.7)	−1.3 (−6.0 to 0.1)
Lobular	−0.7 (−1.1 to -0.2)	−0.30 (−1.0 to 0.02)

This table indicates the average uptake from both scans for FDG and FLT as well as the percent and unit change between pre- and post-therapy scans

### Association between pathology and imaging results

**Fig. 5:**
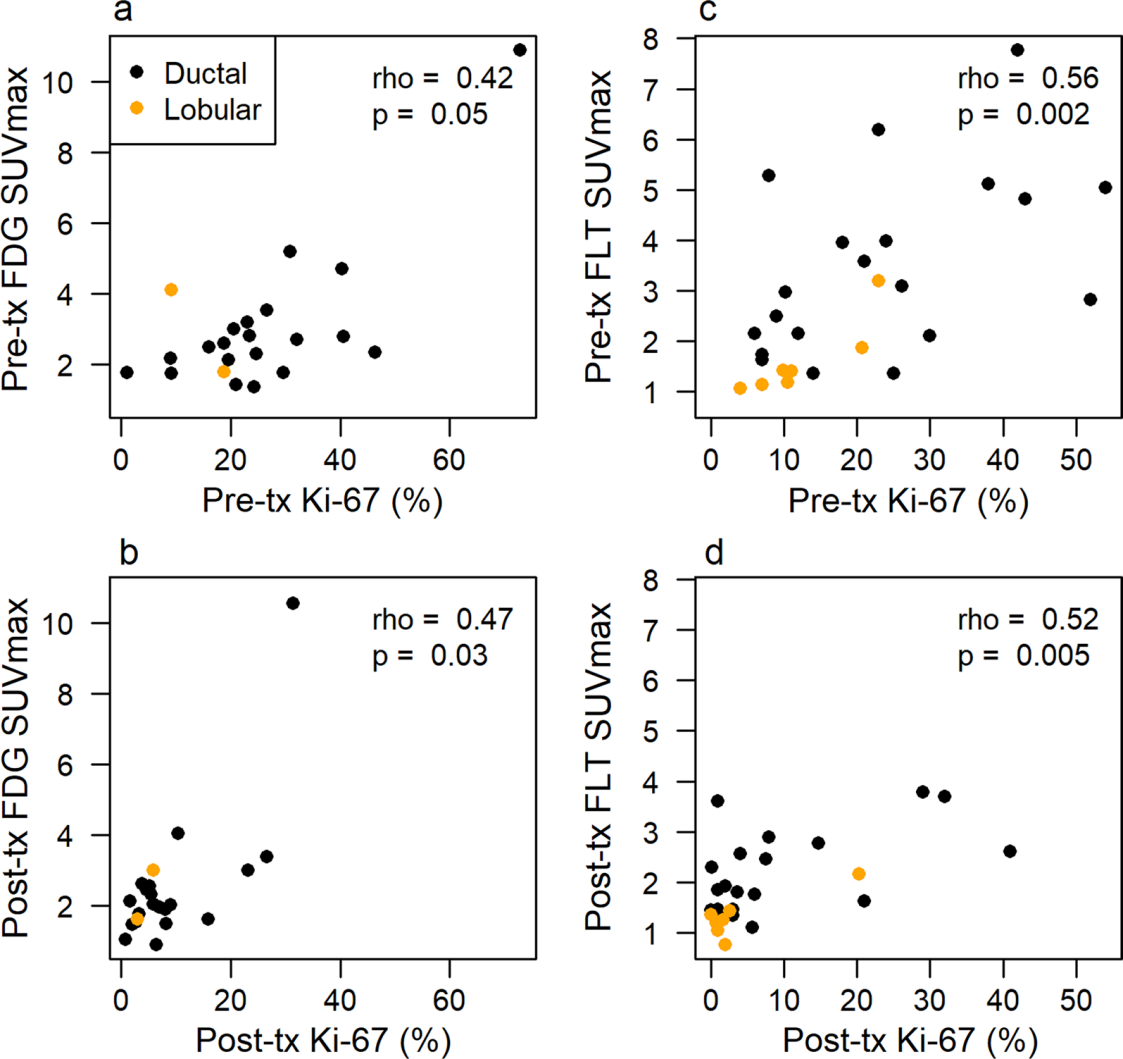
Association between imaging and tissue measures. Pre-therapy **a** FDG or **c** FLT SUVmax and pre-therapy Ki-67 index. Post-therapy **b** FDG or **d** FLT SUVmax and post-therapy Ki-67

Figure 5 illustrates the association between SUVmax and Ki-67% for both studies. Figure 5a, b demonstrates the association between FDG uptake measures and Ki-67% at baseline and post-therapy (surgery), with a combined Spearman rank order correlation coefficient of 0.55 (*p* < 0.001). The association between FLT uptake measures and Ki-67 at baseline and post-therapy is shown in Fig. 5c, d. The combined Spearman rank order correlation coefficient is 0.60 (*p* < 0.001).

**Fig. 6:**
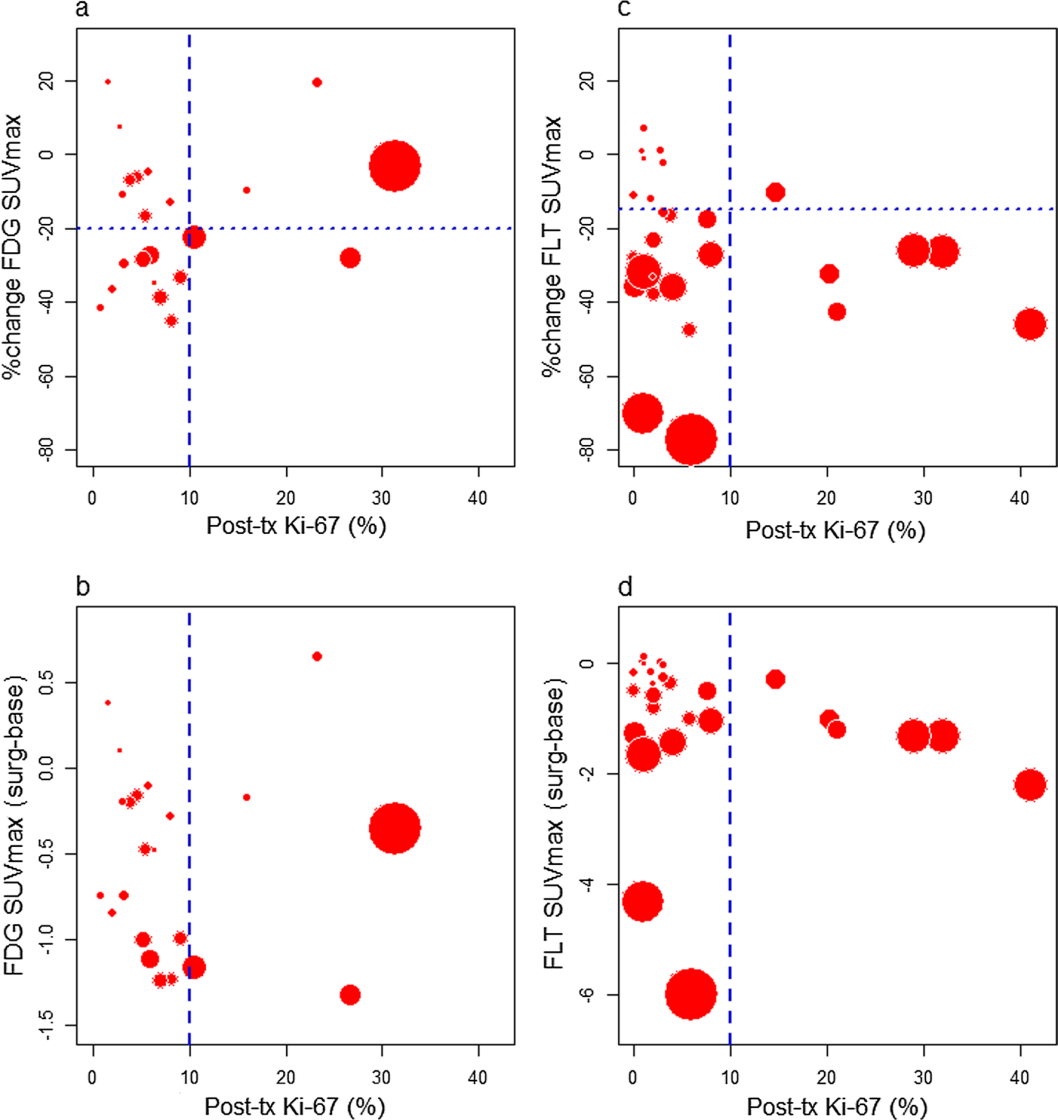
Percent and absolute change in FDG and FLT SUVmax, and post-therapy Ki-67. Vertical dashed line at pre-specified Ki-67 threshold of 10%; horizontal dotted lines at pre-specified thresholds for response as defined in the Methods. Plotting symbols diameters are proportional to uptake of the PET tracer at baseline. With concordance of %change in SUVmax and post-therapy Ki-67, all data points would appear in the lower left and upper right quadrants. The lesions that do not follow this pattern (upper left and lower right quadrants) are not restricted to those with very low baseline uptake

Figure 6 illustrates the percent and absolute change in FDG and FLT SUVmax imaged between baseline and post-therapy Ki-67 using plotting characters with size proportional to baseline PET uptake. Of the 17/22 (77%) tumors with post-therapy surgical specimen Ki-67 values ≤ 10%, 8 (47%) showed metabolic response by FDG (20% or greater decrease in SUVmax, Fig. 6a). Of the 5/22 (23%) of patients with surgery Ki-67 values > 10%, 2 (40%) were classified as metabolic responders. FDG SUVmax metabolic response was not associated with low surgical Ki-67 (mid-*p* = 0.83). There was no rank-order correlation between percent change in FDG SUVmax and surgical Ki-67 (*ρ* = 0.05, *p* = 0.82) (Fig. 6a), or SUV unit change and surgical Ki-67 (*ρ* = 0.26, *p* = 0.20) (Fig. 6b).

Of the 21/27 (78%) tumors with post-therapy Ki-67 values ≤ 10%, 14 (67%) show imaging response by FLT (Fig. 6c). Of the 6/27 (22%) patients with surgery Ki-67 values > 10%, 5 (83%) were classified as imaging responders. Change in FLT SUVmax was not associated with surgical Ki-67, by binary classification of imaging and Ki-67 response (mid-*p* = 0.27) or by continuous values of surgical Ki-67 with percent change in FLT SUVmax (*ρ* = 0.21, *p* = 0.29). The rank-order correlation between surgical Ki-67 and absolute change in FLT SUVmax suggested that greater decrease in FLT SUVmax was associated with higher surgical Ki-67 (Fig. 6d, *ρ* =  −0.41, *p* = 0.03 unadjusted for ad hoc analysis).

### Sensitivity to treatment response analyses and alternate uptake measures

There was no association between AI exposure time and percentage change of FDG or FLT measures (Additional file 1: Fig. S1), between duration of endocrine therapy and Ki-67 response (Additional file 1: Fig. S2) or between the change in SUVmax and the change in Ki-67 index for either FDG or FLT (Additional file 1: Fig. S3).

Given the impact of the small size of many of the lesions, the SUVmax uptake was corrected for partial volume bias and showed no significant differences from SUVmax uptake. Details regarding these methods and results are shown in Additional file 1: Tables S1 and S2 and Additional file 1: Figs. S4–S6.

Similarly, Additional file 1: Table S3 and Additional file 1: Figs. S7–S9 demonstrate correlations for kinetic model data (K_i_). FDG K_i_ by dynamic imaging and a pre-specified 20% cutoff showed a slightly better association with surgical Ki-67 ≤ 10% than pre-treatment FDG K_i_ and Ki-67.

## Discussion

This study shows that it is feasible to monitor patients and measure change in tumor metabolic activity with serial PET imaging during neoadjuvant endocrine therapy to assess for early response in vivo. The majority of tumors manifest a decline in uptake beyond what would be expected for the established reproducibility of the imaging test; specifically, 50% (95% CI 31–69%) of patients studied by FDG-PET and 70% (95% CI 52–84%) of patients studied by FLT-PET. We noted a statistically significant association between both PET imaging measures and Ki-67 values both pre- and post-therapy (Fig. 5), noting that Ki-67 after a short exposure to endocrine therapy has been shown to have predictive value for long-term response [12]. Taken together, these data indicate promise for both PET tracers as imaging biomarkers of the impact of endocrine therapy on tumor proliferation, with a narrower confidence interval for FLT, as expected by the tighter correlation to proliferation.

In both studies, we found a correlation between baseline and pre-operative uptake and proliferation measures in tissue. Recent studies of serial FLT in breast cancer patients undergoing neoadjuvant chemotherapy showed good correspondence between post-therapy uptake and Ki-67 and were predictive of response [19, 38]. The value of post-therapy FDG-PET has also been shown for PET imaging studies of breast cancer [16, 23, 39, 40].

Our hypothesis that change in FDG or FLT uptake between baseline and pre-surgery would predict endocrine response based on post-therapy Ki-67 values was not confirmed (Fig. 6). Response as defined by post-therapy Ki-67 does not show perfect concordance with imaging response, as it did for our pilot study with FDG-PET imaging in patients with advanced disease [23], but is similar to observations of endocrine therapy impact by others [21]. One reason for the discrepant results could be the small tumor size in this study. However, our analysis with partial volume correction also did not reveal an association (Additional file 1: Fig. S5). Another possibility is that indolent tumors with low pre-therapy uptake of tracer might respond differently to therapy [41]. However, tumors with Ki-67 response that lacked response by imaging included both indolent and metabolically active tumors (Fig. 6). We also used a two-tissue compartment model to test dynamic measures of tracer flux, which has been shown to provide greater sensitivity to uptake changes in response to therapy [41]. The dynamic measures did not reveal the expected association between imaging changes and post-therapy Ki-67, which suggests changes in these radiotracers’ uptake in tumors may be measuring changes in different underlying biological mechanisms than those assessed by changes in Ki-67 values (Additional file 1: Fig. S9).

We prospectively defined imaging response as a 20% decline in FDG-PET [35] and 15% for FLT based on prior published test/re-test data for these tracers [36]. Low pre-therapy tracer uptake as well as low Ki-67, present in many patients in this study, likely impacted our findings, especially the ability to measure changes in uptake by SUV [41]. Within the FDG cohort, 8 patients were classified as metabolic non-responders but had a Ki-67% < 10% at surgery. All of these patients had baseline and pre-surgery SUVmax values of ≤ 3.

Limitations of our studies include a relatively small number of patients and variability in duration of AI therapy; this was due to patient convenience sampling. We obtained the tissue and imaging around the time of the surgical resection. It is possible that treatment lasting longer than the planned 2-week window confounded comparison between PET measures and Ki-67 tissue assays; however, the intervals we encountered are typical of clinical practice and the duration of therapy did not appear to influence the magnitude of change in PET measures or Ki-67 (Additional file 1: Figs. S1–S2). Moreover, in the IMPACT trial, the Ki-67 drop noted at two weeks persisted at 12, suggesting that the decline in tumor proliferation endured at a similar level in patients remaining on therapy [11]. Both cohorts contained samples of ductal and lobular lesions. Patients with lobular cancers responded well to endocrine therapy, as one would anticipate with endocrine sensitive tumors. There were too few patients with lobular disease, however, to draw any conclusions, although these tumors did not appear to differ significantly from ductal tumors (Figs. 2, 3, 5 and Additional file 1: Figs. S4, S5, S7, and S8). Both pre- and post-menopausal patients were included in the study. While this makes the group potentially more heterogeneous, the therapy was identical, and favorable baseline characteristics similar. Enrolled patients all had operable tumors, and baseline Ki-67 did not differ by menopausal status (*p* = 0.3, Wilcoxon rank-sum test). Our findings suggest that both pre- and postmenopausal women’s tumor response can be successfully measured with FDG- and FLT-PET.

Endocrine therapy is powerful treatment for ER+ breast cancer, alone or in synergy with other therapies. In clinical practice, genomic assays routinely determine which patients merit chemotherapy in addition to endocrine therapy [42]. FDG- and FLT-PET are complementary tools to tissue assays that hold promise to measure early tumor changes to indicate sensitivity in vivo. Several recent and ongoing studies are looking at Ki-67 to stratify which patients require chemotherapy. An in vivo marker of similar response could avert the need for biopsy and allow whole tumor measures of response. FDG and FLT-PET are promising to detect changes in tumor biology early, prior to shrinkage of tumor, and could be used to measure the impact of CDK4/6 inhibitors, increasingly used with endocrine therapy in ER+ breast cancer [25], or other molecularly targeted agents. FDG-PET is commonly used clinical practice and has a favorable biodistribution for both primary tumors and metastases, is Food and Drug Administration (FDA) approved, and routinely available, but does not directly measure cellular proliferation. FLT, on the other hand, is a validated tracer of cellular proliferation, but is investigational, and while it is well visualized in breast and regional nodal lesions, it has high liver and bone marrow uptake, making its application to metastatic breast cancer more challenging. As other novel tracers are in development, PET imaging can help to evaluate molecularly targeted agents and allow patients to remain on neoadjuvant treatment safely for a longer duration to then achieve a measurable pathologic response at surgery, determine which patients could avoid chemotherapy, and/or which patients will benefit from endocrine therapy alone [26, 43].

## Conclusions

Serial FDG- and FLT-PET imaging are feasible following a short course of AI therapy in a pre-operative, window-of-opportunity setting. While the change in tracer uptake was not predictive of post-therapy Ki-67 for either tracer, the pre- and post-therapy uptake correlated well with pre- and post-therapy Ki-67 values for both FDG and FLT. Although more studies are needed, these results suggest that imaging pre-therapy and after short exposure to endocrine therapy, or perhaps just at one time point after starting therapy, may provide clinically useful data to help guide breast cancer treatments.

## Supplementary Information

**Additional file 1.** Supplemental Material.

## Data Availability

These data are shared on clinicaltrials.gov (NCT01928186).

## Abbreviations

FDG: ^18^F-fluorodeoxyglucose
FLT: ^18^F-fluorothymidine
PET: Positron emission tomography
AI: Aromatase inhibitors
ER+: Estrogen receptor positive
SUVmax: Maximum standardized uptake value
HER2: Human epithelial growth factor receptor 2
GnRH: Gonadotropin releasing hormone
IND: Investigation new drug
MRI: Magnetic resonance imaging
CT: Computerized tomography
VOI: Volume-of-interest
IHC: Immunohistochemistry
PR: Progesterone receptor
FDA: Food and Drug Administration
