# Supplementary material for: 18F-fluorodeoxyglucose (FDG) PET or 18F-fluorothymidine (FLT) PET to assess early response to aromatase inhibitors (AI) in women with ER+ operable breast cancer in a window-of-opportunity study
Source: Breast Cancer Res. 2021 Aug 23;23:88. doi: 10.1186/s13058-021-01464-1 (PMC8381552; doi:10.1186/s13058-021-01464-1)
Supplement: Supplementary file 1 — Additional file 1. Supplemental Material. [file 13058_2021_1464_MOESM1_ESM.pdf]

Supplemental Material

Serial ^{18}F-Fluorodeoxyglucose (FDG) PET or ^{18}F-Fluorothymidine (FLT) PET assess early response to aromatase inhibitors (AI) in women with ER+ operable breast cancer in a window-of-opportunity study

Perrin E Romine[1], Lanell M Peterson[1], Brenda F Kurland[2], Darrin W Byrd[3], Alena Novakova-Jiresova[4], Mark Muzi[3], Jennifer M Specht[1], Robert K Doot[5], Jeanne M Link[6], Kenneth A Krohn[6], Paul E Kinahan[3], David A Mankoff[5], and Hannah M Linden[1]

[1]Division of Medical Oncology, University of Washington, Seattle, WA;
[2]University of Pittsburgh, Pittsburgh, PA;
[3]Department of Radiology, University of Washington, Seattle, WA;
[4]Department of Oncology, First Faculty of Medicine, Charles University and Thomayer Hospital, Prague, Czech Republic;
[5]Department of Radiology, University of Pennsylvania, Philadelphia, PA;
[6]Department of Diagnostic Radiology, Oregon Health and Science University, Portland, OR;

Corresponding Author:
Hannah M Linden, M.D.
Division of Medical Oncology
University of Washington/Seattle Cancer Care Alliance
ORCID 0000-0001-5535-746X
825 Eastlake Ave E, Seattle, WA 98109
Email: hmlinden@uw.edu

Supplemental Table 1. Additional study methods

**Imaging Characteristics-GE PET/CT**	
Slice thickness (mm)	3.27
Reconstruction diameter (mm)	550
Array size (pixels)	128x128
Low dose CT	60 mA, 2.5mm
Injection duration	1 minute
Dynamic Sequence FLT	16x5s, 7x10s, 5x30s, 5x1m, 5x3m, 7x5m
Dynamic Sequence FDG	4x20s, 4x40s, 4x1m, 4x3m, 8x5m
Reconstruction Method	2D FBP
Convolution kernel (filter, mm)	Hanning, 7
Scatter correction method	Convolution Subtraction
**FLT quality control**	
Radiochemical purity	≥95%
Chemical purity	<0.61 µg/ml per injected dose
Specific activity	>160 Ci/mmol

SUV_{max} calculation methods:

To calculate SUVmax, using the 30-60 min summed images constructed from the dynamic data, square volume-of-interest (VOIs) of 3x3 pixels were drawn on identified lesions over three consecutive slices encompassing the pixels with the most uptake. The pixel with the most uptake was used to calculate SUV_{max} as shown below:

$$SUVmax = \frac{Max\ Tissue\ Activity\ (kBq/cc)}{\frac{Injected\ dose\ (MBq)}{Body\ Weight\ (kg)}} \quad \text{Eq.1}$$

Body weight SUVs are reported as dimensionless under the assumption that 1 mL of imaged tissue weighs 1 gram.

Partial Volume Correction methods:

Lesion size was measured on contrast-enhanced lesions from a clinical MRI scan done close to the time of the first PET scan. PV corrections were based on previously calculated size-dependent recovery coefficients (RC) derived from phantom data and applied to the SUVmax values [1]. Briefly, to correct

for the partial volume effect, we used contrast recovery coefficients measured with a National Electrical Manufacturers' Association Image Quality phantom that was filled with long-lived 68Ge/68Ga nuclide and epoxy. The phantom contained six spheres of diameters 10, 13, 17, 22, 28, and 37 mm. These were filled at 4 to 1 contrast relative to the background. Fifty PET images with independent image noise were reconstructed from 5-minute acquisitions using the same reconstruction settings as the clinical images. The max signal from regions of interest placed on the spheres were computed to characterize signal bias versus feature size. Contrast recovery coefficients (CRCs) were computed using the known sphere signal value *T*, known background *B*, measured maximum sphere value *t* and measured background value *b*. The formula was

$$CRC = \frac{t-b}{T-B} \qquad \text{Eq. 2}$$

and was computed for each sphere size. After averaging over the 50 independent images, cubic spline interpolation was used to generate CRCs for all lesion sizes between 0 and 38mm. For lesions larger than 38 mm, the correction factor for 38 mm was employed. The curves were forced to go through the origin to reflect the expectation that contrast recovery should vanish for very small contrast values.

Corrected SUVmax values (SUVpvmax) were then computed by first subtracting the background signal intensity as measured in the contralateral breast, scaling the signal-above-background, and adding the background back in. That is the lesion signal $\tilde{S}$ was computed as

$$\tilde{S} = \frac{S-b}{CRC} + b \qquad \text{Eq. 3}$$

where S is the measured SUVmax and b is the measured background in the breast [2]. The CRC was selected from the interpolated curves using lesion size determined from MR images.

Model parameter K_i (Flux) methods:

Dynamic imaging and kinetic modeling was done as previously described for both FLT and FDG [3-7]. Briefly, the VOIs drawn on the 30-60 minute summed images were applied to the dynamic image set. An approximately 1cc VOI was also drawn over the left ventricle to create the blood input function. Two-tissue compartment models were utilized to calculate the kinetic parameters using PMOD version 3.6 (Zurich, Switzerland). Metabolic flux (K_i), was estimated from parameters derived by fitting the input function and the blood-activity curve to the tissue time-activity curve data, and calculated as follows:

$$Ki = \frac{K1k3}{k2+k3} \qquad \text{Eq. 4}$$

K1 represents the transfer of blood into tissue; k2 is the transport back to blood; and k3 represents metabolic trapping of the tracer.

Supplemental Table 2. FDG and FLT PET imaging results for SUVpvmax

	FDG study	FLT study
PET measure	Mean (range)	
SUVpvmax (pre-therapy)		
All	3.5 (1.3-10.3)	3.3 (1.2-7.3)
Ductal	3.6 (1.3-10.3)	3.8 (1.5-7.3)
Lobular	3.0 (1.9, 4.0)	2.0 (1.2-3.0)
SUVpvmax (post-therapy)		
All	2.9 (1.4-10.0)	2.2 (0.8-4.2)
Ductal	2.9 (1.4-10.0)	2.4 (1.2-4.2)
Lobular	2.3 (1.7, 2.9)	1.6 (0.8-2.6)
SUVpvmax (percent change)		
All	-17% (-45 to 28%)	-26% (-77 to 7%)
Ductal	-17% (-45 to 28%)	-30% (-77 to 7%)
Lobular	-19% (-27, -11%)	-15% (-33 to 1%)
SUVpvmax (unit change)		
All	-0.6 (-1.7 to 0.9)	-1.1 (-5.6 to 0.1)
Ductal	-0.6 (-1.7 to 0.9)	-1.4 (-5.6 to 0.1)
Lobular	-0.6 (-1.1, -0.2)	-0.4 (-1.0 to 0.02)

Supplemental Table 3. FDG and FLT PET imaging results for model K_i (flux), (mL/min/g x 10^3) = μL/min/g

	FDG study	FLT study
PET measure	Mean (range)	
K_i flux (pre-therapy)		
All	6.2 (<0.05-55.2)	24.2 (0.8-62.6)
Ductal	6.4 (<0.05-55.2)	28.0 (0.8-62.6)
Lobular	4.2 (<0.05, 8.3)	13.3 (2.0-36.5)
K_i flux (post-therapy)		
All	4.9 (<0.05-53.2)	15.7 (1.5-35.2)
Ductal	4.9 (<0.05-53.2)	17.1 (1.5-35.2)
Lobular	4.4 (3.1, 5.7)	11.4 (2.6-26.4)
K_i flux (percent change)		
All	-18% (-99% to 100%)	-17% (-82% to 100%)
Ductal	-22% (-99% to 100%)	-21% (-82% to 100%)
Lobular	18% (-63%, 100%)	-3% (-74% to 100%)
K_i flux (unit change)		
All	-1.4 (-7.8 – 5.6)	-8.6 (-51.6 – 34.3)
Ductal	-1.5 (-7.8 – 1.7)	-10.9 (-51.6 – 34.3)
Lobular	0.2 (-5.2, 5.6)	-1.9 (-10.1 – 9.1)

Supplemental Fig. 1 Association between AI exposure time and percent change of (a) FDG or (b) FLT measures

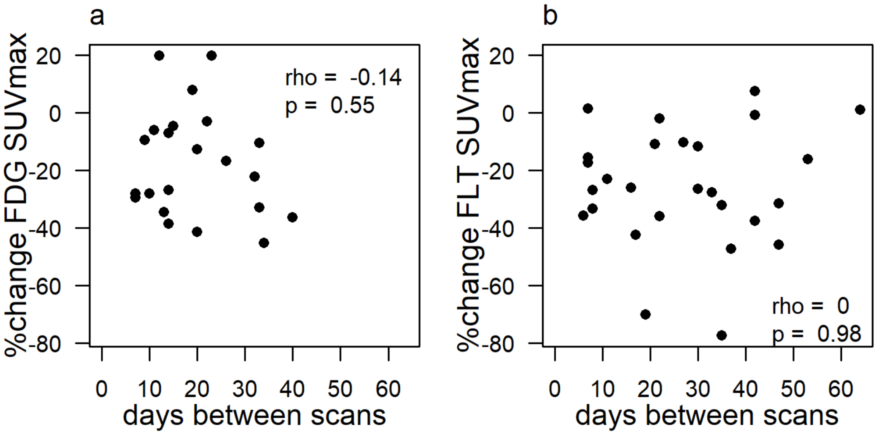

Supplemental Fig. 2 Association between duration of endocrine therapy and Ki-67 response. (a) FDG study and (b) FLT study

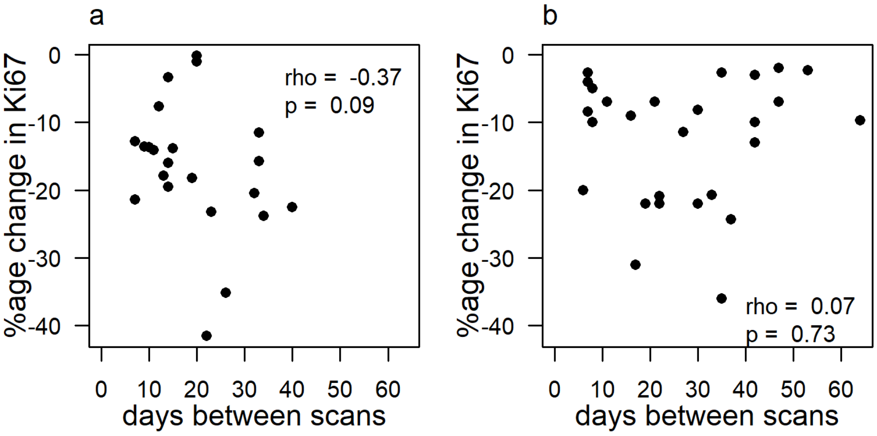

Supplemental Fig. 3 Association between change in SUVmax and the change in Ki-67. (a) FDG study and (b) FLT study

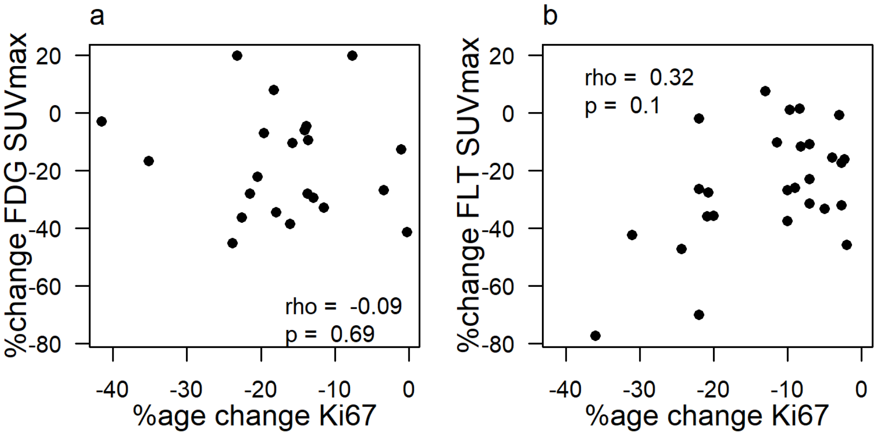

Supplemental Fig. 4 Pre-treatment and post-treatment measures (a) FDG SUVpvmax (b) FLT SUVpvmax shown as days on AI therapy.

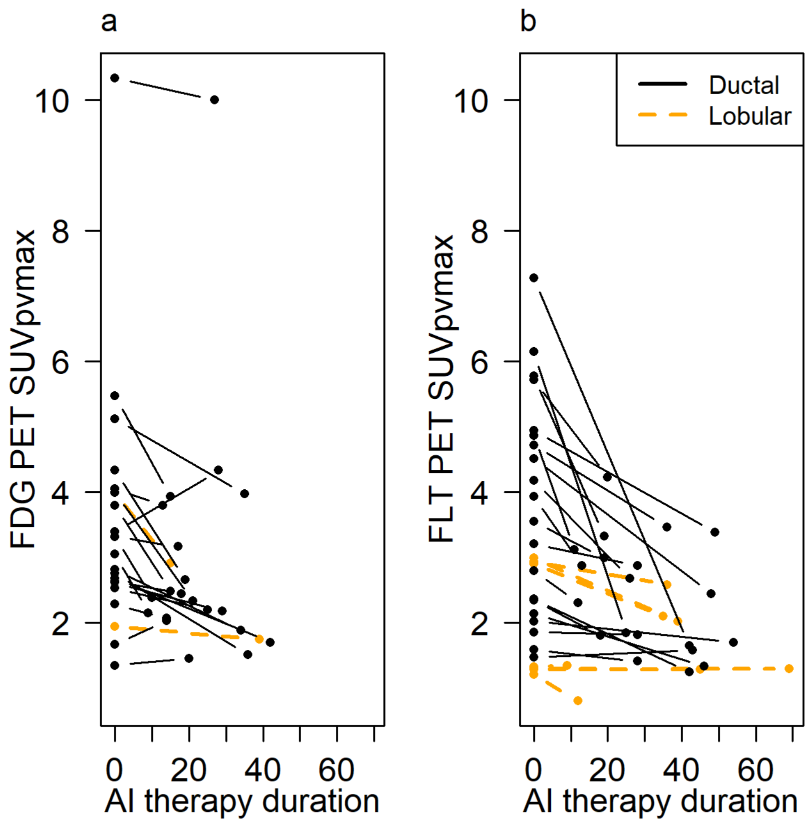

Supplemental Fig. 5 Association between imaging and tissue measures. Pre-therapy (a) FDG or (c) FLT SUVpvmax and pre-therapy Ki-67 index. Post-therapy (b) FDG or (d) FLT SUVpvmax and post-therapy Ki-67

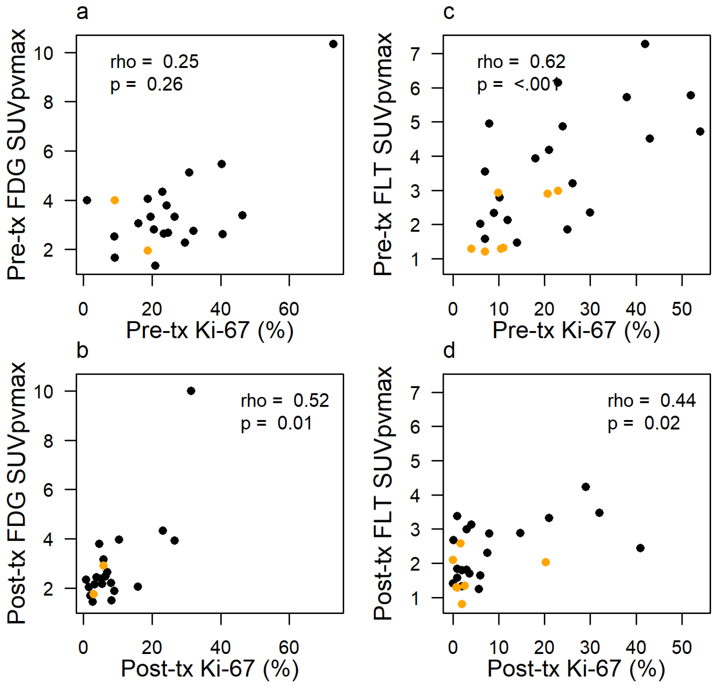

Supplemental Fig. 6 Association between imaging and tissue measures. Percent change in (a, c) and absolute change (b, d) FDG/FLT SUVpvmax and post-therapy Ki-67

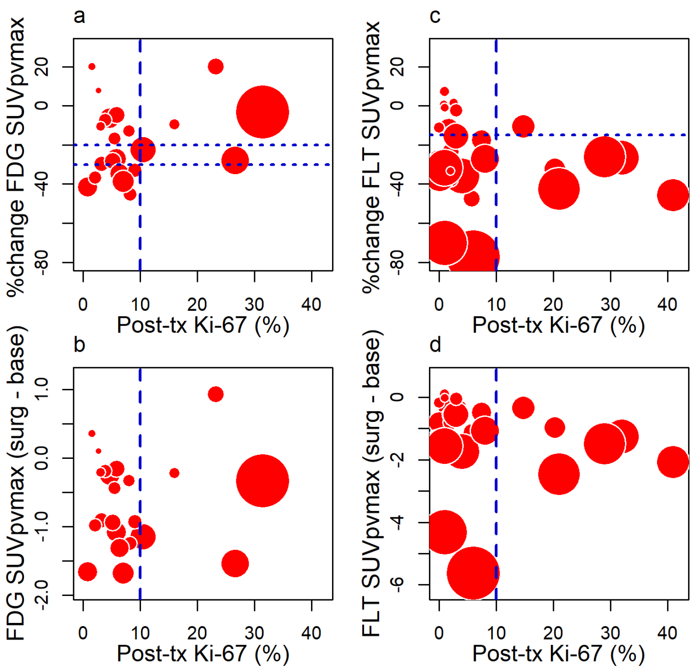

Supplemental Fig. 7 Pre-treatment and post-treatment measures (a) FDG K_i (flux) (b) FLT K_i (flux) µL/min/g shown as days on AI therapy.

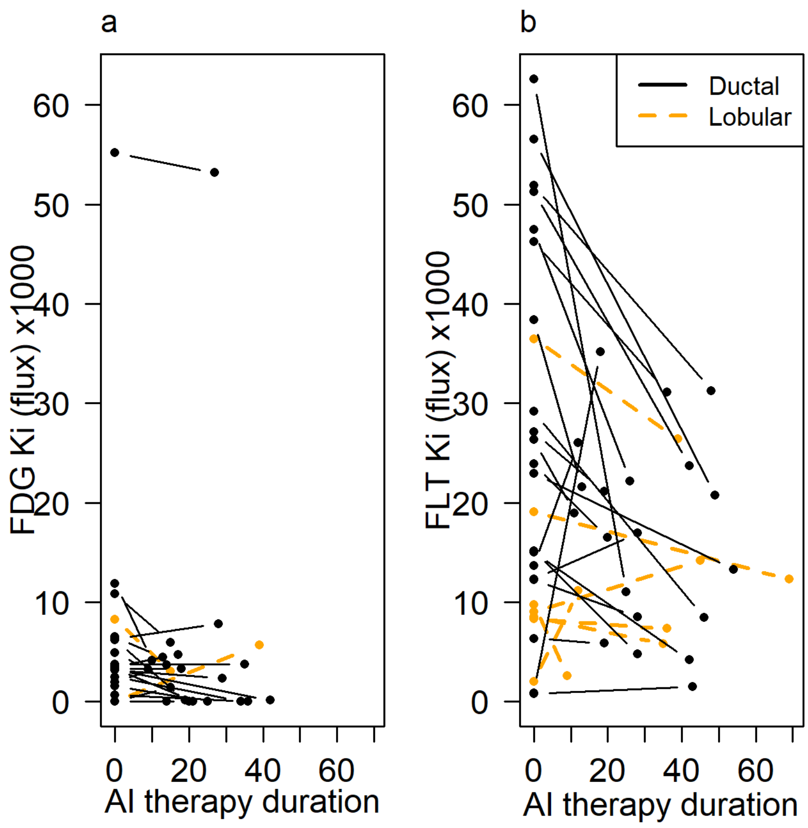

Supplemental Fig. 8 Association between imaging and tissue measures. Pre-therapy (a) FDG or (c) FLT K_i (flux) and pre-therapy Ki-67 index. Post-therapy (b) FDG or (d) FLT K_i (flux) and post-therapy Ki-67

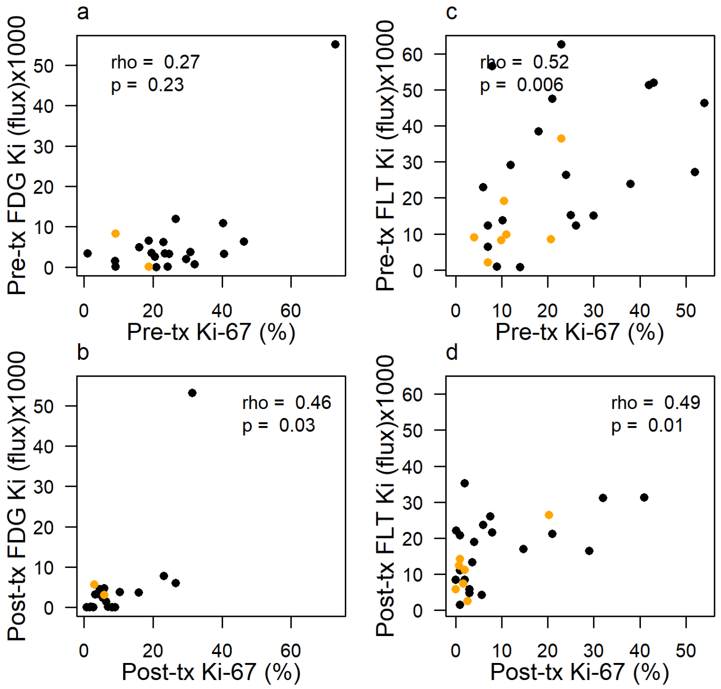

Supplemental Fig. 9 Association between imaging and tissue measures. Percent change (a, c) and absolute change (b, d) FDG/FLT K_i (flux) and post-therapy Ki-67, with floor of -50% and ceiling of +20% change in K_i (flux) µL/min/g

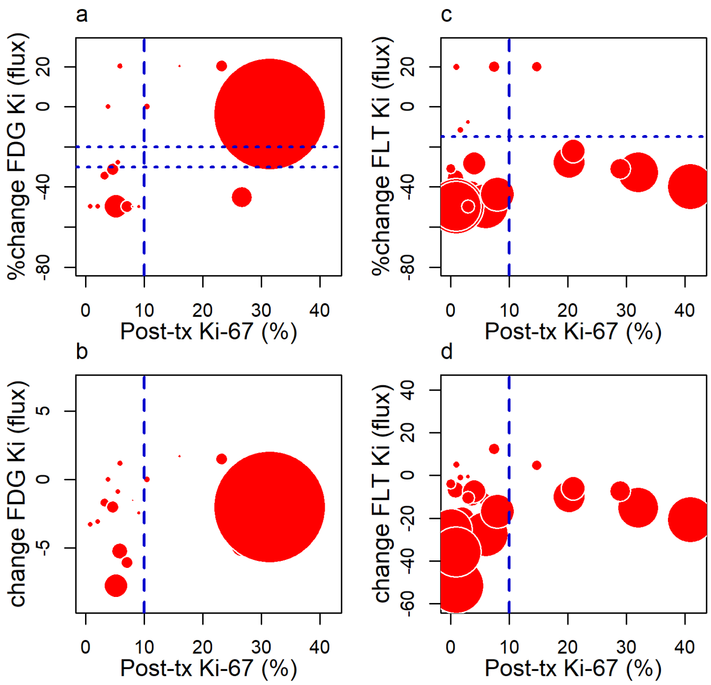

REFERENCES

1. Kessler RM, Ellis JR, Jr., Eden M. Analysis of emission tomographic scan data: limitations imposed by resolution and background. J Comput Assist Tomogr. 1984;8(3):514-22.
2. Peterson LM, Mankoff DA, Lawton T, Yagle K, Schubert EK, Stekhova S, et al. Quantitative imaging of estrogen receptor expression in breast cancer with PET and 18F-fluoroestradiol. J Nucl Med. 2008;49(3):367-74.
3. Mankoff DA, Dunnwald LK, Gralow JR, Ellis GK, Charlop A, Lawton TJ, et al. Blood flow and metabolism in locally advanced breast cancer: relationship to response to therapy. J Nucl Med. 2002;43(4):500-9.
4. Mankoff DA, Dunnwald LK, Gralow JR, Ellis GK, Schubert EK, Tseng J, et al. Changes in blood flow and metabolism in locally advanced breast cancer treated with neoadjuvant chemotherapy. J Nucl Med. 2003;44(11):1806-14.
5. Muzi M, Mankoff DA, Grierson JR, Wells JM, Vesselle H, Krohn KA. Kinetic modeling of 3'-deoxy-3'-fluorothymidine in somatic tumors: mathematical studies. J Nucl Med. 2005;46(2):371-80.
6. Muzi M, Spence AM, O'Sullivan F, Mankoff DA, Wells JM, Grierson JR, et al. Kinetic analysis of 3'-deoxy-3'-18F-fluorothymidine in patients with gliomas. J Nucl Med. 2006;47(10):1612-21.
7. Muzi M, Vesselle H, Grierson JR, Mankoff DA, Schmidt RA, Peterson L, et al. Kinetic analysis of 3'-deoxy-3'-fluorothymidine PET studies: validation studies in patients with lung cancer. J Nucl Med. 2005;46(2):274-82.
